# The Anti-Inflammatory Mediator, Vasoactive Intestinal Peptide, Modulates the Differentiation and Function of Th Subsets in Rheumatoid Arthritis

**DOI:** 10.1155/2018/6043710

**Published:** 2018-08-01

**Authors:** Raúl Villanueva-Romero, Irene Gutiérrez-Cañas, Mar Carrión, Selene Pérez-García, Iria V. Seoane, Carmen Martínez, Rosa P. Gomariz, Yasmina Juarranz

**Affiliations:** Departamento de Biología Celular, Instituto de Investigación Sanitaria Hospital 12 de Octubre (imas12), Universidad Complutense de Madrid, Madrid, Spain

## Abstract

Genetic background, epigenetic modifications, and environmental factors trigger autoimmune response in rheumatoid arthritis (RA). Several pathogenic infections have been related to the onset of RA and may cause an inadequate immunological tolerance towards critical self-antigens leading to chronic joint inflammation and an imbalance between different T helper (Th) subsets. Vasoactive intestinal peptide (VIP) is a mediator that modulates all the stages comprised between the arrival of pathogens and Th cell differentiation in RA through its known anti-inflammatory and immunomodulatory actions. This “neuroimmunopeptide” modulates the pathogenic activity of diverse cell subpopulations involved in RA as lymphocytes, fibroblast-like synoviocytes (FLS), or macrophages. In addition, VIP decreases the expression of pattern recognition receptor (PRR) such as toll-like receptors (TLRs) in FLS from RA patients. These receptors act as sensors of pathogen-associated molecular pattern (PAMP) and damage-associated molecular pattern (DAMP) connecting the innate and adaptive immune system. Moreover, VIP modulates the imbalance between Th subsets in RA, decreasing pathogenic Th1 and Th17 subsets and favoring Th2 or Treg profile during the differentiation/polarization of naïve or memory Th cells. Finally, VIP regulates the plasticity between theses subsets. In this review, we provide an overview of VIP effects on the aforementioned features of RA pathology.

## 1. An Introduction to the Physiopathology of Rheumatoid Arthritis

Rheumatoid arthritis (RA) is a systemic inflammatory disease mainly manifested with peripheral polyarthritis. Genetic background, epigenetic modifications, and environmental factors are considered the aetiological factors that could cause an inadequate immunological tolerance towards critical self-antigens, characteristic of all autoimmune diseases [1, 2]. As consequence, chronic inflammation in the joints and an imbalance between different Th subsets are triggered in this pathology [2, 3]. Altered oral, intestinal, or lung microbiota, such as *Porphyromonas gingivalis*, *Aggregatibacter actinomycetemcomitans*, *Proteus mirabilis*, *Prevotella copri*, or *Mycoplasma fermentans*, and other infectious agents such as EpStein-Barr virus interfere with the equilibrium between inflammation and tolerance [4–8]. They can trigger the pathology through molecular mimicry by means of two important characteristics, the generation of autoantibodies such as anticitrullinated protein antibodies (ACPA) or rheumatoid factor and the inflammatory response [1, 2].

The first step in the activation of an inflammatory response is the recognition of two kinds of ligands by specific receptors named PRRs. In the initial stages, they recognize the conserved molecular structures expressed by invading microbes named pathogen-associated molecular patterns (PAMPs). The uncontrolled inflammation in chronic diseases leads to tissue damage generating endogenous danger-associated molecular patterns (DAMPs) including stress signals such as damaged or apoptotic cells [9–11], representing the second kind of ligands recognized by PRRs [11]. The stimulation of these receptors produces the expression of proinflammatory cytokines such as tumor necrosis factor (TNF)*α*, interleukin- (IL-) 6, IL-1*β*, or IL-12 by several innate cells such as dendritic cells (DCs) or macrophages, which link innate and adaptive immunity. Inflammation in RA patients is maintained by sustained activation of multiple inflammatory positive-feedback regulatory pathways in a variety of cells [12]. As a consequence of all these processes that have been unleashed in early RA, there is an alteration in the balance between the different subpopulations of Th lymphocytes. Paracrine and autocrine actions of cytokines, along with persistent adaptive immune responses, can perpetuate the disease and ultimately lead to cartilage and bone destruction in the joints [1].

Naïve CD4^+^ T helper cells can differentiate into Th1, Th2, Th17, Th9, Th22, follicular helper T (Tfh), and regulatory T (Treg). Th subset differentiation is orchestrated by complex regulatory networks that allow for shared transcriptional programs and plasticity across T cell subsets [13, 14]. Among all subtypes of Th cells, Th17 are the most heterogeneous and plastic subsets [13–15]. The heterogeneity of Th phenotypes generated in the primary response is mirrored in the heterogeneity of memory Th cells that persist once the pathogen has been eliminated [16]. This heterogeneity of Th cells serves to target the cells to the tissues where they are needed and to define the class of immune and tissue response that is appropriate for the type of pathogen attack. However, some of these subpopulations have been related to a greater or lesser extent with the malfunctions of the immune system, specifically with autoimmune diseases, like RA. About half of infiltrated cells in the synovial sublining in established RA are Th cells and contribute directly along with macrophage-like synoviocytes (MLS), fibroblast-like synoviocytes (FLS), and other adaptive immune cells to the damage in cartilage and bone due to synovial invasion into adjacent articular structures, being a cardinal sign of RA [1].

Prior to the discovery of Th17 cells in 2005, Th1 was the subset involved in RA pathology showing an imbalance between Th1 and Th2 subsets [17]. Nowadays, Th17 cells have acquired a main role in the pathogenesis of RA [2, 15, 18, 19]. This subset is heterogenic and can show a pathogenic or nonpathogenic profile, depending on the cytokine balance present in the microenvironment during its differentiation/activation. The cytokines that produce each one are different. Pathogenic Th17 cells express RORC, the transcription factor characteristic for Th17 cells, as well as the transcription factor characteristic for Th1, T-bet. Thus, this pathogenic Th17 cells produce, in addition to IL-17 or IL-21 (cytokine characteristic of Th17 subset), IFN*γ*, IL-22, GM-CSF, and other proinflammatory cytokines [20]. Memory Th17 cell cultures “ex vivo” from early RA patients show more pathogenic profile than Th17 cells from healthy donors [19, 21]. It was revealed that these pathogenic Th17 cells can shift to Th1 cells (named “ex-Th17” or “nonclassical Th1” cells), which are reported to be more pathogenic than Th17 cells per se in RA [15, 17, 19, 22]. These ex-Th17 or nonclassical Th1 cells are accumulated in the joints of rheumatoid arthritis patients and can explain the observation that the therapeutic strategies against IL-17 are not sufficient in RA [22, 23]. The hypothesis resides on the “switch” into pathogenic Th17 and nonclassical Th1 at the sites of local inflammation such as the joints [2], promoting GM-CSF production that is the weapon of pathogenic Th17 cells and one of the novel therapeutic target in RA [24–26].

CD4^+^CD25^+^FoxP3^+^ T cells (Treg) can suppress other immune cells by regulating their proliferation and cytokine production. No differences were detected in the number of total Treg and bona fide Treg subsets (CD45RA^+^FoxP3^low^ naïve Treg, CD45RA^−^FoxP3^high^ activated Treg, and CD45RA^−^FoxP3^low^ non-Treg) from seropositive arthralgia patients compared to healthy donors [27]. Whether Tregs are functionally different is still unknown [14]. Nonpathogenic Th17 cells are closely related with Treg, expressing transcription factor FoxP3 and producing IL-10 among others [20, 28]. In RA patients, the transdifferentiation of Th17 from Treg contributes to perpetuation of RA during anti-TNF treatment [29].

CD4^+^CXCR5^+^ T cells, follicular helper T cells or Tfh, collaborate with B cells to produce antibodies and are closely related to the other Th subsets. Differential expression of CXCR3 and CCR6 within CD4^+^CXCR5^+^ T cells defines three major subsets: CXCR3^+^CCR6^−^ (Tfh1), CXCR3^−^CCR6^−^ (Tfh2), and CXCR3^−^CCR6^+^ (Tfh17) [30]. RA patients, both with active disease and in remission, demonstrate an increased frequency of Tfh and overrepresentation of Tfh subsets bearing a B cell helper phenotype [30, 31]. There are few studies conducted in RA regarding the Th22 subpopulation; nevertheless, its characteristic cytokine, IL-22, which can also be produced by pathogenic Th17, has shown to have a pathogenic role associated with disease activity in RA, promoting osteoclastogenesis and bone destruction in RA [32]. The percentage of CD4^+^IFN*γ*^−^IL-17^−^IL-22^+^ (Th22) cells in RA patients was markedly increased comparing with healthy donors and positively correlated with DAS28 [33]. With respect to the last subset, Th9, although with lesser importance than the others, Th9 cells and IL-9 were frequently detected in peripheral blood mononuclear cells and synovia of RA patients [34].

### 1.1. A Panoramic View of VIP's Role in Rheumatoid Arthritis

It is important to bear in mind that RA is a dynamic disease in its development, so a complete understanding of how RA develops over time is important to set up therapies that prevent disease progression rather than treating its symptoms [35]. The importance of the neuroimmune network in joint homeostasis has been shown. Indeed, several neuropeptides identified in joint tissues, including vasoactive intestinal peptide (VIP), have been suggested to have a role as neuroosteological regulators in bone metabolism. VIP is a homeostatic and immunoregulatory peptide involved in the control of both innate and adaptive immune response. It has only one chain of 28 amino acids, with some residues crucial to its functions, which sequence is highly conserved during evolution. It belongs to the secretin/glucagon family of peptides which share an *α*-helix structure [36]. This neuropeptide can act locally or systematically as it is produced by sympathetic nerve endings, lymphocytes, or even FLS in the joint [37, 38]. VIP is involved in a broad range of functions through its binding to its specific G-protein-coupled receptors, VPAC_1_ and VPAC_2_ [39]. Healing effects of exogenous administration of VIP in animal models of inflammatory/autoimmune diseases have been described; specifically, VIP prevents arthritis in a CIA model through its anti-inflammatory and immunomodulatory actions [40, 41]. In humans, “ex vivo” effects of VIP have been demonstrated in lymphocytes, macrophages, and FLS [21, 38, 42]. In summary, VIP is a microenvironment mediator capable of modulating all the stages mentioned above in RA from the arrival of pathogens to the differentiation of Th cells. It exerts a direct antimicrobial activity against a variety of pathogens [43, 44] and modulates TLR expression in several cells, even in FLS from RA patients [45–47]. In addition, VIP decreases proinflammatory mediators in lymphocytes and FLS from RA patients [21, 38, 48–50] and modulates the differentiation of several Th cells from RA patients, including a decrease in the pathogenic profile and plasticity of some of them [21, 50, 51]. In addition to the role of VIP in “ex vivo” samples from RA patients, endogenous VIP also plays a major role in patients with RA, allowing to stratify patients with early RA for therapeutic decision making in the “window of opportunity” [52]. VIP gene polymorphisms, associated with its serum levels, predict treatment requirements in early rheumatoid arthritis [43, 53]. Indeed, lower levels of its receptor, VPAC_1_, in PBMCs are associated with more severe inflammation and higher disease activity in RA patients [54].

Taking all this into consideration, this review provides a deep description of the role of this anti-inflammatory mediator, VIP, in the differentiation and function of Th subsets in rheumatoid arthritis, capable to modulate all the stages between the arrival of pathogens and the differentiation of Th cells in RA.

## 2. Infectious Agents in Rheumatoid Arthritis and Effect of VIP as Antimicrobial Mediator

In recent years, the significance of the role played by alterations in microbiota and infections has deepened our understanding on the significance of this process in triggering RA.

One of the very first insights about the relation between RA and infection came hand to hand with the increased risk of arthritis associated with periodontal disease [55]. This association is thought to be partly mediated by oral bacteria members' microbiota such as *Porphyromonas gingivalis* [56] or *Aggregatibacter actinomycetemcomitans* [57]. These two bacteria are capable of triggering autoimmunity in RA by means of molecular mimicry, the former, and through the production of leukotoxin A and the subsequent NETosis process, the latter. In RA patients, it has been observed that *P. gingivalis*, via PADI4 (peptidyl arginine deiminase type IV), causes an aberrant citrullination of proteins inducing loss of tolerance to citrullinated peptides [58, 59] providing a link between the infectious process and the autoimmune response. An alternative form of action for these bacteria consists of its possible binding to the PRR toll-like receptor 2 (TLR-2), increasing the production of interleukin-1 (IL-1) and consequently stimulating the differentiation of T cells into the T helper 17 (Th17) subpopulation [60]. In this context, it has been reported that in early arthritis patients a prompt periodontitis treatment could avoid the development of a chronic and progressive arthritis [61].

Besides oral microbiota, gut microbiota could also play an important role in arthritis. In rodent studies, it has been observed that susceptibility and severity of arthritis decreased significantly when maintained in a germ-free environment or in the presence of restricted bacterial flora [62, 63], suggesting that microbiome can exert helper functions increasing the autoimmune process of the pathology. In patients with RA, it has been described an alteration in the gut microbiota compared to healthy population. Specifically, it has been found a decreased diversity of abundant commensal taxa such as Bifidobacteria and Bacteroides species [64], parallel to an expansion of *Mycoplasma fermentans*, *Proteus mirabilis* [65], and rare taxa such as *Actinobacteria (Collinsella, Eggerthella)* [66]. Moreover, intestinal levels of *Prevotella copri* are higher in recent onset RA patients than in healthy donors or in established RA patients, being a possible marker of early disease [7]. Both in the case of oral and intestinal microbiome, it has been observed that the alterations found in patients with active RA are restored, at least in large part, as a result of immunosuppressive treatment [67].

Lung mucosal microbiota has lately been involved in the development of RA as infectious agents, such as *Streptococcus pyogenes* and *S. pneumoniae*, both pulmonary pathogens, are combined with smoking in causing a breakdown in immune tolerance [68]. There is an increased incidence of ACPA in smokers and also in RA-related lung disease [69, 70]. The prevalence of oral mucosa infections in RA patients is high [71], but RA patients without these infections might have other bacterial organisms as disease initiators. For instance, it has been described a lung dysbiosis in RA patients similar to sarcoidosis patients which could be involved in the inflammatory process present in RA pathology [72].

The hypothesis that an infectious event could be involved in RA induction has been taken into consideration for a long time [4, 73], even though to date there is no clearly identified causative pathogen nor a direct association with a specific infection. In recent years, integrated theories have emerged with force to clarify the RA etiopathogenesis. In these theories, together with genetic predisposition and environmental factors, the mucous membrane of the lungs, the oral cavity, and the gastrointestinal tract, could have a leading role, given that they are the interface between external influences and the immune system. Thus, RA, probably, could originate far from the joints. As we have previously pointed out, a plausible explanation could be that stressful events in these tissues lead to posttranslational modifications of peptides with the consequent formation of autoantibodies directed against them. These antibodies generated in the mucous membranes could be transferred throughout the body through the bloodstream, converting the local autoimmunity process into systemic autoimmunity. Upon reaching the joint, they could find additional antigens, unleashing local inflammatory events in the synovium with the consequent activation of different cell types (fibroblasts, macrophages, osteoclasts, etc.). Thus, activating the production of proinflammatory cytokines would lead to synovitis, persistent inflammation, and destruction of the bone and cartilage [74–76].

A recent study carried out in mice showed how microbial infection at mucosal sites is able to provoke a break in tolerance leading to the generation of autoreactive and antipathogen T cells, predominantly, Th17 cells [77]. In addition to Th17 cells, other subsets of T cells could be regulated by gut microbiota in autoimmune processes. Block et al. have found that follicular helper T cells (Tfh) are critically important for the development of arthritis in the K/BxN autoimmune arthritis model. Besides, Tfh cell differentiation is modulated by the microbiota [78]. Although further studies in humans are still required, these results point to the capability of infections in triggering autoimmunity beyond the epitope mimicry and the autoreactive T cell activation.

Regarding other infectious agents, Epstein-Barr virus (EBV) infection has been classically associated with RA [74], and a recent study has shown a strong relation between the Chikungunya virus infection and the development of polyarthralgia [79].

**Figure 1:**
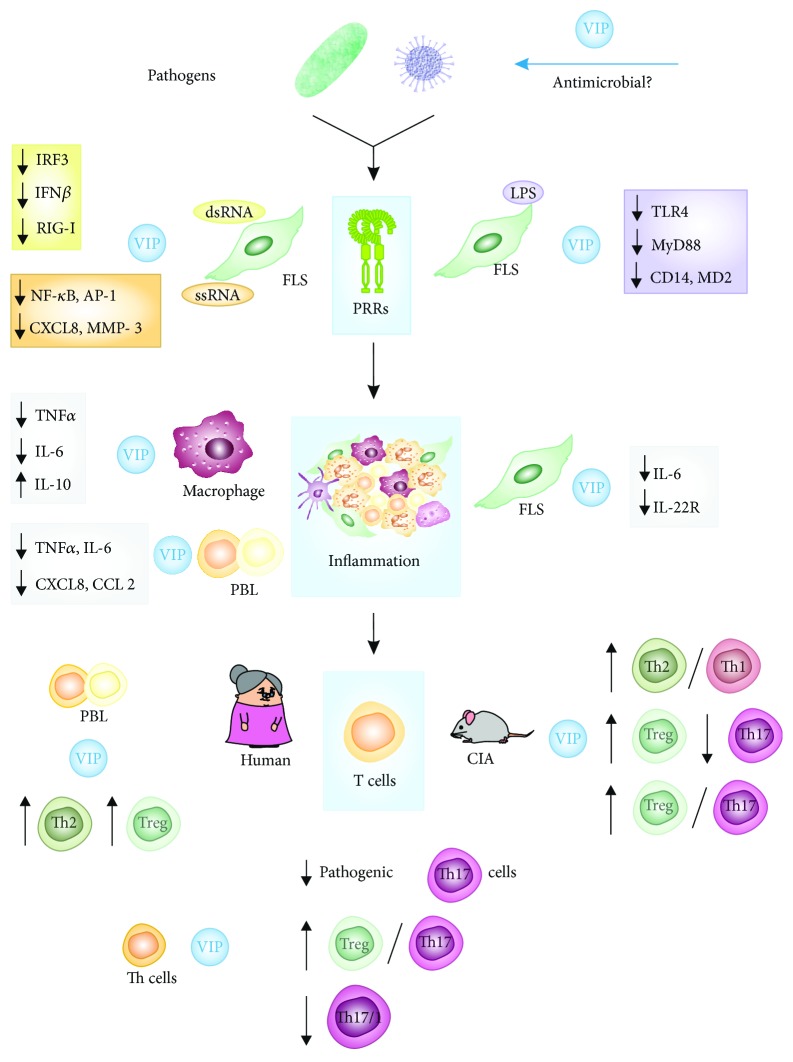
Effect of VIP on RA. VIP is a microenvironment mediator capable of modulating all the stages in RA from the arrival of pathogens to the differentiation of Th cells. It exerts a direct antimicrobial activity against a variety of pathogens, modulates TLR expression and function in several cells, decreases proinflammatory mediators in lymphocytes and FLS, and modulates the differentiation of several Th cells, including a decrease in the pathogenic profile and plasticity of some of them. PRRs: pattern recognition receptors; FLS: fibroblast-like synoviocytes; PBL: peripheral blood lymphocytes; CIA: collagen-induced arthritis mouse model.

Although the study of the role of infections and microbiota on RA in humans still need more profound research, the role of VIP regulating pathogen microbes at different levels is noteworthy. Young animal fed with prebiotic-enriched milk showed higher ileal VIP expression and lesser relative abundance of pathogenic microbes, such as *Collinsella* [80]. It has been recently reported that this peptide exerts a direct antimicrobial activity against a wide range of bacteria, including *S. aureus*, *E.coli*, or *P. aeruginosa* [43] as well as against the African trypanosome *T. brucei* [81]. VIP is also protective in polymicrobial sepsis and cutaneous leishmaniasis [44], and its release has been described under microbial-induced inflammation [82]. Moreover, VIP decreases induced responses by LPS from *P. gingivalis* on monocytes [83] (Figure 1). The awareness of this ability added to the anti-inflammatory and immunomodulatory properties of this peptide opens new possibilities in its clinical application against infectious diseases. In summary, removing inflammatory stimuli rooted in infectious foci, specially in the intestine and mouth, could achieve the inhibition of the chronic immune loop, and thereby improvements in the arthritis pathogenic process could be attained.

## 3. Effect of VIP on Functions Mediated by Pattern Recognition Receptors in Rheumatoid Arthritis

Immune system function is mainly based on discriminating between self and non-self, allowing recognition and removal of parasites and pathogens ranging from the smallest viruses and bacteria to the largest multicellular parasites. This capacity is dependent on the presence of PRRs. These receptors are classified into three types namely, NOD-like receptors (NLR), RIG-like receptors (RLR), and toll-like receptors (TLR). NLR are implicated in the modulation of inflammatory and apoptotic responses; RLR are related to intracellular recognition of RNA virus replication, and TLR are involved in warning the immune system against extracellular or endosomal PAMPs [84, 85]. Expression of these receptors is a hallmark of the innate immune system, and activation of this branch of immunity leads to the effective priming of adaptive immune responses mediated by B and T cells. These cells exhibit receptors for antigens and, by means of education and cooperation, are able to distinguish self from non-self-antigen and trigger subsequent actions. Thus, defects in the coordinated action of both innate and adaptive systems as well as in the recognition of self-antigens by the adaptive immune system are the root cause of autoimmune diseases. Specifically, TLR are the main players in the self-non-self-discrimination, and it is well demonstrated that deficiency in the TLR-mediated recognition of self-antigens triggers many autoimmune disorders including rheumatoid arthritis (RA) [86, 87]. In fact, RA seems to result from an autoimmune dysfunction in its early stages that later progresses to chronic inflammation of the synovial joints [10]. Besides the abovementioned hypothesis of the altered microbiota, which postulates a systemic origin of RA where PRRs probably play a relevant role, potential ligands for these receptors have been identified in the joint microenvironment of RA patients, including PAMPs such as peptidoglycans, bacterial DNA, and viral double-stranded RNA (dsRNA) or single-stranded RNA (ssRNA) [88–90]. Moreover, it has also been reported the presence of host-derived mRNA, heat-shock proteins, fibronectin, and apoptotic and necrotic cellular debris, which could contribute to RA perpetuation [91, 92]. Once exogenous or endogenous ligands initiate TLR signaling, Toll/IL-1 receptor (TIR) domain binds to TIR domain-containing adaptor proteins like myeloid differentiation primary-response protein 88 (MyD88) or TIR domain-containing adaptor protein inducing IFN*β* (TRIF). Following complex and tightly regulated signaling pathways, TLR stimulation finally leads to the downstream activation of JNK, p38 MAPKs, and transcription factors namely NF-*κ*B and IRFs. These transcription factors induce the synthesis and secretion of proinflammatory cytokines, type I interferons, and costimulatory molecules [84–86].

TLR are expressed in a variety of cell types in the joint, such as myeloid cells, FLS, T and B cells, osteoclasts progenitors, and endothelial cells. These receptors are distributed into two cell locations, namely plasma membrane (TLR1, TLR2, and TLR4–6) and intracellular compartments (TLR3 and TLR7–9). All TLR, with the exception of TLR6 and TLR10, are present in the synovial tissues and cells from arthritic joints [88], being TLR2 and TLR4 the best characterized and TLR5 and TLR8 the more recently described [93, 94]. In recent years, TLR have been shown to be involved in the pathophysiology of RA, participating in synovial membrane inflammation, osteoclasts maturation, angiogenesis, endothelial function, and in the functional modulation of different subpopulations of T lymphocytes. In this sense, activation of TLR2, TLR3, and TLR4 in FLS from RA patients exacerbated inflammatory Th1 and Th17 cell expansion both in cell–cell contact-dependent and inflammatory cytokine-dependent manner, which induced more IFN*γ* and IL-17 accumulation [10, 95]. Moreover, it has been reported that the engagement of TLR3 can directly induce the synthesis of IL-17A and IL-21 and drive differentiation of human naive CD4^+^ T cells [96].

In particular, MLS and FLS are considered effector cells with an essential role in TLR-mediated inflammatory mechanisms involved in the onset and development of RA [10].

### 3.1. Through the Modulation of Plasma Membrane Receptor TLR4

As we have previously described, in humans, VIP is produced by FLS which also expressed its receptors VPAC_1_ and VPAC_2_. Likewise, VIP expression is downregulated both in RA-FLS and in TNF*α*-treated FLS, suggesting that a minor presence of this endogenous anti-inflammatory factor may contribute to the pathogenesis of RA [38]. It is worth to mention that resident FLS represent important players in RA synovitis initiation and propagation, releasing proinflammatory mediators, such as chemokines, that support inflammatory cell retention and chronification of the disease.

On the basis of the abovementioned and given that FLS express several members of the TLR family, our studies have focused on the effects of VIP on TLR regulation in FLS from RA patients. We have described the expression of TLR2 and TLR4 in RA FLS which exhibited increased levels of TLR4 transcripts and protein compared with FLS from osteoarthritis (OA) patients (Figure 1). Besides, VIP treatment decreased the LPS- and TNF-induced expression of TLR4 and MyD88 in FLS from RA patients, reducing downstream responses such as the production of CCL2 and CXCL8 chemokines [97]. Moreover, VIP also modulated TLR signaling pathways by downregulating the LPS-induced overexpression of both CD14 and MD2, coreceptors required for TLR4 signaling, as well as molecules of the MyD88-dependent and MyD88-independent ways. VIP reduced gene expression of several downstream molecules of MyD88-dependent pathway, including different kinases. Regarding MyD88-independent pathway, VIP also downregulated the transcripts of TRIF and TRAM adaptor molecules involved in the engagement of adaptive immunity. As an aside, VIP treatment led to an impaired production of IL-6 and RANTES/CCL5 after LPS stimulation, demonstrating the functional significance of VIP effect on mediators derived from MyD88-dependent and MyD88-independent pathways, respectively [46, 98]. Taken together, these results demonstrate that VIP acts as a negative modulator of TLR4 signaling, regulating the production of several pivotal molecules of the pathway (Figure 1).

### 3.2. Through the Modulation of the Functions of Intracellular Compartment Receptors, TLR3 and TLR7

We have also studied the effect of VIP in FLS after TLR3 or TLR7 stimulation by dsRNA and ssRNA analogous, respectively, by studying the transcription factors involved and the subsequent effects on antiviral IFN*β*, proinflammatory CXCL8 chemokine, and matrix metalloproteinase 3 (MMP-3). Results showed that VIP was not able to diminish the expression of these receptors but significantly reduced the dsRNA-induced IRF3 nuclear translocation and consequently the production of IFN*β*. Concerning TLR7 signaling, VIP significantly decreased activation of every transcription factor (NF-*κ*B, AP-1 c-Jun, and AP-1 c-Fos) on ssRNA-treated FLS, leading to a subsequent decrease in CXCL8 and MMP-3 production, which plays an important role in joint destruction [47] (Figure 1).

### 3.3. Through the Modulation of RLR Receptors

RLR are cytosolic pattern recognition receptors that comprise helicase retinoic acid-inducible gene I (RIG-I) and melanoma differentiation-associated gene 5 (MDA5) which recognize dsRNA. The constitutive and dsRNA-induced expression of RIG-I and MDA5 has been described in FLS from RA patients, and our results have shown that VIP is able to inhibit significantly the dsRNA-induced gene expression of RIG-I [47] (Figure 1).

## 4. VIP as Anti-Inflammatory Peptide in Rheumatoid Arthritis

In addition to the decrease in inflammatory TLR signaling, the anti-inflammatory action of VIP has been described extensively in several animal models treated with exogenous VIP or in the “ex vivo” treatment of human or murine macrophages and T lymphocytes [37, 42, 99, 100]. Delgado et al. first published its anti-inflammatory role in RA in 2001 [40, 101]. This study demonstrated that VIP treatment dramatically suppresses clinical joint disease in murine collagen-induced arthritis (CIA) regulates adaptive and innate immune responses. Its pleotropic actions have a salutary effect on both inflammation and immunity in the CIA model.

In human RA pathology, the effect of VIP as anti-inflammatory peptide was confirmed in synovial tissue cells and FLS from RA patients, where VIP downregulated chemokine production and IL-6 more clearly after stimulation with TNF*α* [102]. In FLS, VIP decreased IL-22-specific receptor and prevented the contribution of rheumatoid synovial fibroblasts to IL-22-mediated joint destruction [48]. This anti-inflammatory role of VIP has been detected in other immune cells in RA, such as macrophages or peripheral blood lymphocytes (PBL) from RA patients cultured “ex vivo” [42, 50]. The presence of this neuropeptide decreased the levels of proinflammatory mediators for instance TNF*α*, IL-6, CXCL8, and CCL2 after polyclonal stimulation with PMA/ionomycin in PBLs from RA patients [50]. Besides, VIP decreases TNF*α* and IL-6 while it augments IL-10 in macrophages polarized to imitate those presented in the inflamed joint [42] (Figure 1).

Although its anti-inflammatory role has been widely described, occasionally, we observed no effect or an opposite effect, especially with no inflammatory stimulus in resting cells, suggesting that this peptide may exhibit dual actions depending on the activation status of the cell. By the way, the presence of VIP when blood cells from RA patients were cultured in the absence of activators or stimulators increased some proinflammatory cytokines or decreased IL-4 production [50, 103], showing a homeostatic function in these cells.

## 5. VIP Is Involved in the Generation of Diversity/Plasticity of Th Subpopulations in Rheumatoid Arthritis

In addition to its anti-inflammatory potential, Delgado et al. first described the capacity to modulate subpopulations of Th lymphocytes [40]. VIP suppresses Th1 cell function and differentiation, as demonstrated by the decreased IFN*γ* expression. On the other hand, Th2 function is enhanced in VIP-treated mice, as determined by the observed increase in IL-4 production. In the era of Th1/Th2 dichotomy, it was thought that this peptide possibly “rebalanced” these Th subsets in the immune system [40, 101]. This fact was also observed in other animal models of autoimmune diseases such as Crohn's disease, multiple sclerosis, and autoimmune diabetes [37, 104] regardless of the way of administration since “*in vivo”* delivery of lentiviral vectors expressing VIP complementary DNA had the same effect than intraperitoneal administration of VIP [105]. In this animal model of RA, VIP not only shifted the immune response towards a Th2-type response but also expanded CD4^+^CD25^+^ Treg in the periphery, which inhibited autoreactive T cell activation/expansion [106]. After Th17 discovery, it has also demonstrated that this neuropeptide also downregulates Th17 response in CIA model, increasing the balance between Treg/Th17 subsets and influencing in this way on the RANK/RANKL/OPG system [41, 107, 108] (Figure 1).

These studies with exogenous administration of VIP in animal models of RA were completed using samples “ex vivo” of RA patients. In PBL from RA patients, the presence of VIP increased the levels of IL-4 and IL-10 after polyclonal stimulation with PMA/ionomycin, favoring a Th2/Treg profile. Indeed, VIP potentiates Th2 differentiation from healthy donors CD4^+^CD45RA^+^ (naïve T cells) [50]. Our results with activated/expanded memory Th cell “ex vivo” from early RA patients showed that these cells generate a greater proportion of Th17 cells with pathogenic Th17 and Th17/1 profile. VIP lowered this pathogenic profile, decreasing IL-22, GM-CSF, IL-2, IL-21, IL-23R, IL-21R, T-bet, and STAT3, although the effect was higher in healthy donors than in RA patients [21]. These results are in agreement with the fact that VIP maintains the nonpathogenic profile of human Th17 polarized cells, decreasing their Th1 potential [109]. Even when memory Th cells from early RA patients were polarized towards a nonpathogenic Th17 profile in the presence of TGF*β*, they produced significantly more IL-22 and IFN*γ* and show a more Th17/1 profile. The presence of VIP in the conditioned medium reduced IL-22 levels but increased IL-10 and IL-9. Indeed, VIP inhibits Th17 polarization bias to Th1-like cells, inducing a negative correlation between the master regulators for Th1 and Th17 subsets, Tbx21 (T-bet), and RORC (ROR*γ*t), respectively. These data suggest that VIP reduces the pathogenic profile of Th17-polarized cells from early RA patients, increasing Treg/Th17 profile and decreasing Th17/Th1 profile [51]. VIP can exert the Th17 modulation in these cells since both VPAC receptors are present in them; however, an unbalance between VPAC_1_ and VPAC_2_ was observed. The proportion of VPAC_2_ was higher in activated memory Th cells and Th17-polarized cells from early RA patients than from healthy donors [21, 51] (Figure 1).

The ability of VIP to modulate Th17 or Th1 effects in RA is not restricted only to its effect on CD4 T lymphocytes since VIP counteracts the enhancing effect of proinflammatory molecules on IL-22R, IL-17R, and IL-12 family of cytokines in FLS disfavoring the cross-talk between FLS and Th1/Th17 cells [49, 102].

In summary, given the importance of pathogenic Th17 and nonclassical Th1 cells in RA pathology, VIP is shown as a mediator present in the microenvironment, capable of modulating the pathogenicity of Th17 cells and their Th1 plasticity in RA patients.

## 6. Conclusions

VIP modulates all the stages between the arrival of pathogens and the differentiation of Th cells in the pathology of RA through its anti-inflammatory and immunomodulatory actions. It acts as an antimicrobial peptide, decreases PRR expression and its inflammatory signaling, downregulates the production of proinflammatory cytokines and chemokines, and counterbalances Th subsets decreasing pathogenic Th17 cells and their capacity to shift to Th1 profile.

Taking into account, this endogenous peptide represents excellent candidate for the development of multitarget therapeutic strategies that modulate both innate and adaptive immune system.
